# *In vivo* Calcium Imaging of Evoked Calcium Waves in the Embryonic Cortex

**DOI:** 10.3389/fncel.2015.00500

**Published:** 2016-01-06

**Authors:** Mikhail Yuryev, Christophe Pellegrino, Ville Jokinen, Liliia Andriichuk, Stanislav Khirug, Leonard Khiroug, Claudio Rivera

**Affiliations:** ^1^Neuroscience Center, University of HelsinkiHelsinki, Finland; ^2^INSERM U901, Institut de Neurobiologie de la Méditerranée (INMED), Parc Scientifique de LuminyMarseille, France; ^3^Aix-Marseille Université (AMU), UMR S901, Parc Scientifique de LuminyMarseille, France; ^4^School of Chemical Technology, Aalto UniversityEspoo, Finland

**Keywords:** *in vivo* imaging, calcium imaging, cortical development, two-photon microscopy, purinergic receptors

## Abstract

The dynamics of intracellular calcium fluxes are instrumental in the proliferation, differentiation, and migration of neuronal cells. Knowledge thus far of the relationship between these calcium changes and physiological processes in the developing brain has derived principally from *ex vivo* and *in vitro* experiments. Here, we present a new method to image intracellular calcium flux in the cerebral cortex of live rodent embryos, whilst attached to the dam through the umbilical cord. Using this approach we demonstrate induction of calcium waves by laser stimulation. These waves are sensitive to ATP-receptor blockade and are significantly increased by pharmacological facilitation of intracellular-calcium release. This approach is the closest to physiological conditions yet achieved for imaging of calcium in the embryonic brain and as such opens new avenues for the study of prenatal brain development. Furthermore, the developed method could open the possibilities of preclinical translational studies in embryos particularly important for developmentally related diseases such as schizophrenia and autism.

## Introduction

Formation of early neural networks in the mammalian cortex during embryonic stage is a complex process regulated by chemical cues as well as electrical activity. The prevalent view is that spontaneous activity is manifested as calcium transients in the form of independent intracellular fluctuations as well as ensemble activity in the form of propagating intercellular waves (Owens and Kriegstein, 1998; Weissman et al., 2004; Crépel et al., 2007). Intercellular communication at early stage is mainly dependent upon gap junction-based coupling as opposed to synaptic connectivity later during development (Montoro and Yuste, 2004). The synaptic driven type of activity has been reported in the cortex (Garaschuk et al., 2000; Corlew et al., 2004; Lischalk et al., 2009; Conhaim et al., 2010), hippocampus (Leinekugel et al., 1998), retina (Meister et al., 1991), midbrain (Rockhill et al., 2009), hindbrain (Gust et al., 2003; Hunt et al., 2005) and spinal cord (O'Donovan et al., 1998). Other studies have also shown calcium waves in the ventricular zone propagating in radial glia that are generated by the intracellular second messenger inositol-1,4,5-trisphosphate (IP3) which causes the release of calcium from intracellular stores followed by extracellular release of ATP (Weissman et al., 2004). Intracellular calcium transients are known to have a regulatory role in important events of brain development as neuronal proliferation, differentiation and migration (Komuro and Rakic, 1993, 1996; Spitzer, 2006). Whilst in the embryonic cortex calcium waves have been mainly studied in the zone of actively proliferating cells, investigation on coordinated spontaneous activity in newly differentiated neurons is scarce in mammals (Corlew et al., 2004; Crépel et al., 2007; Allène et al., 2008; Allène and Cossart, 2010).

During these early stages of development the embryo is extremely dependent on the interaction with the mother for e.g. hormones, nutrients, salts and importantly oxygen (Fligny et al., 2009; Moisiadis and Matthews, 2014). Despite the advantages of *in vitro* preparations for disclosing the role of intracellular calcium in the maturation of the cerebral cortex, they do not allow the study of the role of mother-embryo interaction on spontaneous calcium transients in the early mammalian embryonic cortex. Importantly, observations of spontaneous activity *in vitro* do not support the existence of correlated activity in rodents earlier than the second postnatal day. However, they do not provide the certainty that the spatial and temporal characteristic of the activity *in vitro* will trustfully reflect the one *in vivo*. The time of appearance of correlated activity is indeed important considering the role of pattern of calcium activity in gene expression as well as the properties of calcium waves in the entraining of immature network through plasticity mediated by e.g., coincident detection. Despite its crucial importance whether primitive forms of spontaneous correlated activity in the embryonic brain exist *in vivo* is not known. The study of embryonic cortical activity *in vivo* has been hampered by the lack of a method that would allow recording under physiological conditions comprising e.g. normal blood oxygenation, heart beat rate as well as the blood of the embryo supplemented with maternal nutrients and hormones.

Here we present a method for *in vivo* two-photon calcium imaging of mouse embryos at gestational stage E13–E15 that are connected to the mother via the umbilical cord, thus preserving a blood-flow that is supplemented by the mother-embryo interface. We could evoke propagating calcium waves under ketamine/xylazine anesthesia using local high-power laser irradiation. These waves were blocked by inhibition of ATP-receptors and could be enhanced by the pharmacological facilitation of intracellular calcium release.

The present data opens the question whether environmental factors affecting the correlated network activity are important in this context. Thus, the method presented could be used as a model for preclinical investigations of drug compounds relevant for developmental disorders in embryos.

## General methods

All animal experiments were approved by the National Animal Experiment Board, Finland.

### Calcium dye injection into the embryos

Pregnant C57bl/6J mice at the 13–15th day of gestation (E13–E15) were anesthetized with intraperitoneal injection of ketamine/xylazine (80/10 mg/kg) and placed on a heating pad at 37°C.

A single 15–20 mm incision was made in the abdomen, the uterine horn exposed and embryos injected ventricularly with Fluo-4AM in loading solution using a capillary glass pipette. The loading solution consisted of 17 μg of calcium dye Fluo-4AM (Molecular Probes) dissolved in 3 μl of 20% F-127 pluronic® acid (Sigma) in dimethyl sulfoxide (DMSO; Sigma) that was then diluted in artificial cerebrospinal fluid (ACSF; in mM: 125 NaCl, 1.25 NaH_2_PO_4_, 2 CaCl_2_, 1 MgCl2, 5 KCl, 20 D-glucose, 10 HEPES) to reach a final concentration 0.4 mM of Fluo-4. 13 mM FastGreen dye (Sigma) was added to the solution for visual injection guidance. Injections were performed with a Picospritzer II, 1–2 μl per embryo depending on the size of the embryo through a glass pipette with a 12 mm long pulled tip that measured ~50 μm in tip diameter (Drummond Scientific) targeting the lateral ventricle. The uterine horn was rinsed with 37°C ACSF every 2 min to prevent hypothermia. After all embryos had been injected with calcium dye the uterine horn was placed back inside the mother and the cavity was sutured. In some experiments suramin (3.7 mM, Tocris Bioscience) was added to the injected solution.

### Immobilization of embryos

Imaging was performed 30 min after surgery in order to achieve sufficient de-esterification of the dye leading to stable fluorescence. In the experiments under ketamine/xylazine anesthesia, top-up doses of 0.25–0.5 were injected every 30–40 min. The mouse was placed on a heating pad under the objective and the abdominal cavity was opened and the uterine horn exposed. A custom-made heating chamber was positioned above the mouse. 10 cm polystyrene plate with a 2 cm diameter circular aperture covered by a polydimethylsiloxane (PDMS) membrane was fit into a heating chamber. The PDMS membrane was bonded to the polystyrene plate through oxygen plasma exposure (on both) and aminopropyltrimethoxysilane treatment of the polystyrene (1% solution in water, 20 min treatment time, rinsing in deionized water). A 15–20 mm long incision in one direction and a 10 mm long in the perpendicular direction were made in the PDMS membrane. The uterine horn was carefully extracted through the incision in the membrane.

The membrane was sealed first with acrylamide glue (Henkel) and then with agarose 4% (Bioline) to avoid liquid leakage through the membrane. The plate was filled with room temperature ACSF. The uterine horn was then carefully cut to expose the embryo in the yolk sac; the yolk sac was cut and the embryo exposed to the ACSF whilst maintaining the umbilical cord connection with the dam. The ACSF level was temporarily lowered and the embryo was glued to a screw-nut along the spinal cord with acrylamide glue, whilst avoiding any direct contact of glue with the head of the embryo. The screw-nut was positioned using Blu-Tack (Bostik) in a dish so that the head of the embryo was facing the objective. The plate was filled again with ACSF and two-photon imaging was performed using a two-photon Olympus FV-1000MPE system through a 25X water-immersion objective (XLPLAN, Olympus).

### Photostimulation and imaging procedure

Image focus was adjusted using mercury lamp illumination. Photostimulation was produced using the standard bleaching routine in Olympus software. A circular area of 20–30 μm in diameter was irradiated with a femtosecond laser (Mai-Tai, Spectra-Physics) at a wavelength of 800 nm at maximum power for a period of 3 s, causing the initiation and spread of a calcium wave as detected by the intensity increase in Fluo-4AM fluorescence. Imaging was performed using the same laser with an acquisition speed of 1.2 fps at a resolution of 512x512. Prior to every stimulation a five frame baseline image was acquired. Fluorescence was collected via a 515–560 nm filter. Blood-flow was monitored before and after imaging session to confirm the proper physiological condition of the embryo by observing shadows of erythrocytes in autofluorescence signal produced in the vicinity of the vessel wall. In some series of experiments caffeine (38.7 mM, Fluka) was dissolved in ACSF together with 13 mM FastGreen (for injection guidance) and injected in the lateral ventricle of the embryo with the same glass pipette as in the calcium dye injection procedure.

### Immunohistochemistry

After the imaging some embryonic brains were fixed in ice-cold 4% paraformadlehyde overnight. The solution was changed next day to 30% sucrose in PBS. Following the 2 days samples were frozen in TissueTek (Sakura) and 30 and 50 μm thick coronal slices were cut at −20°C and collected to Superfrost Plus microscope slides (Thermo Scientific). Immunohistochemical staining was performed according to standard protocol. Primary monoclonal antibodies Tuj1 specific for differentiated neurons (1/500 mouse, Covance) and secondary Alexa 568 (1/800, Molecular probes) were used. Slices were further mounted with ProLong (Life Technologies) for the following fluorescent imaging using Zeiss Imager.M1 Colibri system with LED illumination via 20X objective.

### Image processing and quantitative analysis

Image stacks were stabilized using the ImageJ plug-ins StackReg (Thévenaz et al., 1998) for short recordings of the induced activity and processed using Kalman filter (written by Christopher Philip Mauer) and descriptor-based registration (Preibisch et al., 2010) for longer recordings of spontaneous activity. Overlapping of histological images before and after the immunostaining was performed using UnwarpJ plug-in in ImageJ (Sorzano et al., 2005). Polar transform of the images was performed with the center given as the point of laser stimulus using the plugin Polar Transform (written by Edwin Donnely and Frederic Mothe) in ImageJ software. The transformed image was divided into regions of interest of square shape with a side 70 μm. Analysis was performed for regions of interest up to 400 μm from the stimulus spot to avoid artifacts due to the transformation procedure. For each followed region of interest the intensity profile over time was calculated and the half-maximum was designated as an event time point. Using detected time points a raster plot of events was built.

The wave border was detected as 5% of fluorescence increase over the basal fluorescence in the cell. The number of detected active cells was normalized to the visible area of loaded dye. Wave propagation distance was calculated as the half-maximum wave propagation distance divided by time the wave reached half of the maximum spread distance.

## Results

### *In vivo* two-photon calcium imaging in the embryos connected to the mother

**Figure 1:**
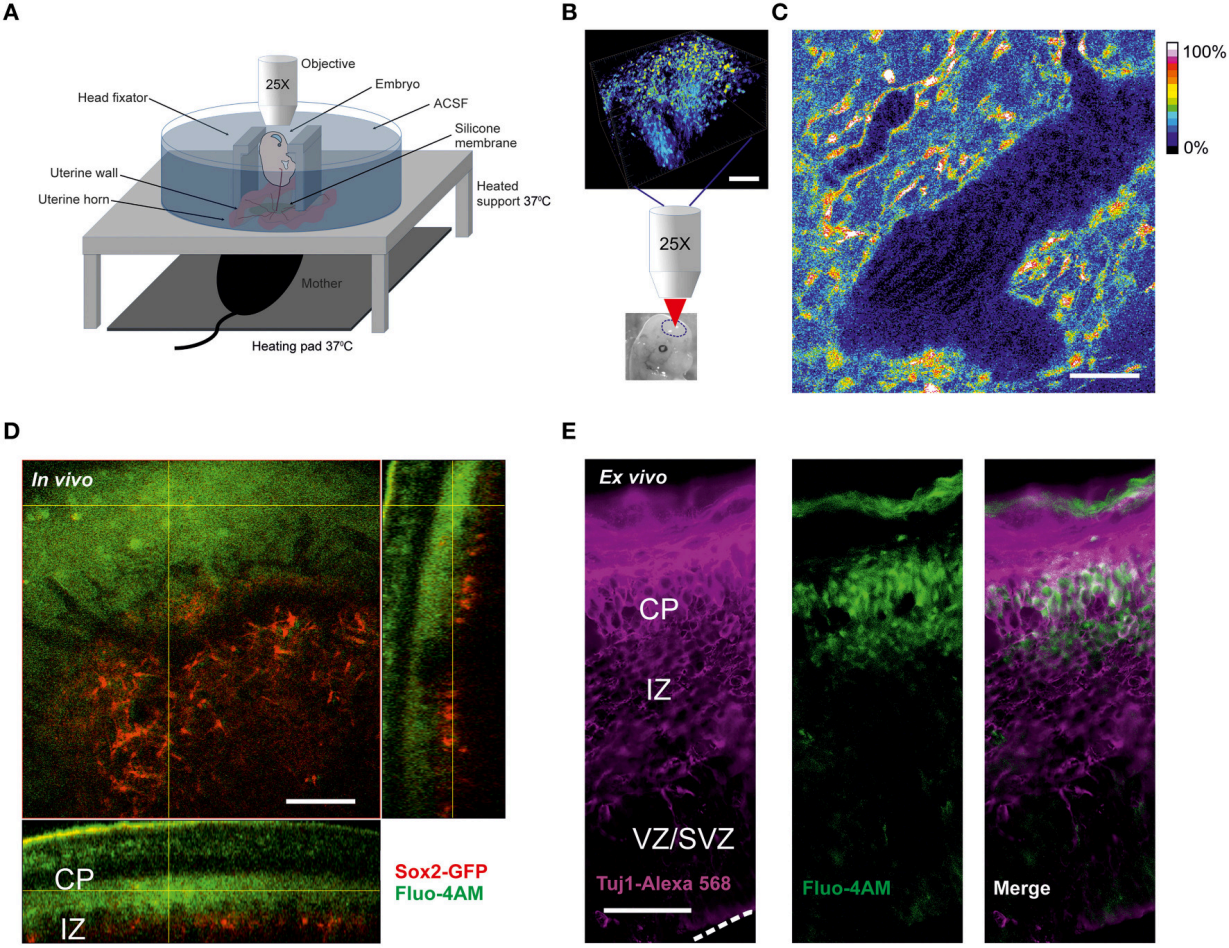
**Calcium imaging in live embryos (A) Scheme of two-photon calcium ***in vivo*** imaging set-up for cortical cells from mice live embryos connected to the anesthetized mother. (B)** Loading of cortical cells with Fluo-4AM calcium dye. Schematic representation of 3D-reconstruction of a z-stack acquired with an axial step of 3 μm following the imaging. The lower image shows the approximate region of imaging in the brain of the embryo shown. Scale bar 100 μm. **(C)** Blood flow visualization by background autofluorescence. The black tilted shadows in the vessels show the moving blood cells. Fluo-4 fluorescence is shown in color-coding. Scale bar 100 μm. **(D)** *In vivo* Fluo-4 calcium dye distribution after intraventricular injection to the Sox2-GFP reporter embryo. Fluo-4 is shown in green (two-photon excitation at 800 nm), GFP-positive cells are shown in red (two-photon excitation at 900 nm), emission is detected through the filter 515–560 nm. Orthogonal view of 3D-reconstruction of a z-stack acquired with an axial step of 3 μm. Scale bar 100 μm. **(E)** Immunohistochemistry for Tuj1 in a cortical 30 μm thick cryosection of embryo cortex after intraventricular injection of Fluo-4AM. Fluo-4 is shown in green (excitation at 375 nm), Tuj1-positive cells are shown in magenta (excitation at 555 nm). Dashed white line delineates the surface of ventricular zone. Scale bar 50 μm.

The study of calcium signaling in the embryonic cortex has been dominated by the *ex vivo* slice preparation with artificial oxygenation and composition of the incubation solution. In order to monitor calcium transients in immature neurons in the conditions of natural oxygenated blood perfusion, we developed a method for imaging live embryos, still connected to the mother by the umbilical cord. To achieve a stable focal plane we used a custom-made recording chamber that offers the advantage of making possible the accessibility of all embryos for imaging with simultaneous control of the animal temperature as well as the liquid environment. The recordings were performed *ex utero* in a modified 10 cm culture plate carrying a central silicone membrane that isolates the embryos without disturbing the blood flow of the umbilical cord (Figure 1A). This approach made it possible to perform experiments on several embryos from the same horn. This procedure also strongly reduced the number of pregnant dams needed for obtaining the same amount of data.

For two-photon calcium imaging of the embryonic brains, cells were initially loaded with calcium dye Fluo-4AM via injection into the ventricular zone prior to imaging (Figure 1B). The use of a membrane-permeant calcium dye has advantages compared to techniques such as micropipette cell loading or expression of genetically encoding calcium indicators by electroporation, in terms of number of loaded cells and versatility of the labeling procedure. However, loading efficiency was dependent on the concentration of the calcium dye, which may be potentially problematic at higher concentration due to nonspecific effects of the dye. We tried different concentrations of the dye in the range 0.2–0.8 mM and we found that 0.4 mM concentration of Fluo-4AM was optimal for *in vivo* imaging. Over the time course of experiment no significant decrease of fluorescence has been observed.

We found that it is possible to perform reliable calcium recordings in the chamber during a period up to several hours on the immobilized E13–E15 embryos since monitoring of heart beating of the mother and the embryos showed that they were not affected by the imaging procedure. During the developmental time window E13–E15 the brain size is optimal for performing intra-cerebroventricular injections because, in addition to the small size of the brain, it is possible to perform two-photon imaging of the entire thickness of the cortex directly through the thin skull (Figure 1B). After E15, the cortex thickens substantially and becomes a limiting factor for deeper layer imaging and also renders difficult the injection procedure. However, images from the surface of the cortical plate can still be obtained post-E15 (data not shown).

A larger volume including the imaged area was scanned after every recording session. The calcium dye loading happened specifically in the area corresponding to the newly forming cortical plate. To ensure that the physiological conditions e.g. oxygenation of the embryos are maintained during the *in vivo* experiment we monitored the blood flow using the autofluorescence signal in the vessels and observed shadows of flowing erythrocytes (Figure 1C). To address the question of which cells type is imaged, we injected Fluo4-AM intraventricularly into the embryos of Sox2-GFP knock-in reporter mouse (D'Amour and Gage, 2003), where GFP is expressed exclusively in the undifferentiated cells. Using a 3D reconstruction of the imaged region in Sox2-GFP reporter (Figure 1D) we could ascertain that the calcium dye was loaded to the cells in the area corresponding to the cortical plate above the Sox2-positive cells. This was also confirmed in the cryosections from *in vivo* loaded brain. The cells loaded with Fluo4-AM dye were predominantly Tuj1-positive (Figure 1E). This characteristic of the AM-calcium dye loading *in vivo* allowed us to record intracellular calcium specifically in the differentiated neurons of cortical plate.

### Propagating calcium waves *In vivo* at the early developmental stage

Previous *in vitro* studies have shown propagation of intercellular calcium waves in radial glia at the embryonic stage of development and in neurons at the postnatal stage. However, the question of what triggers the spontaneous network calcium activity and the properties of these events has not yet been addressed *in vivo*. Moreover, observations of calcium waves in radial glia usually required special non-physiological experimental conditions as low extracellular calcium.

**Figure 2:**
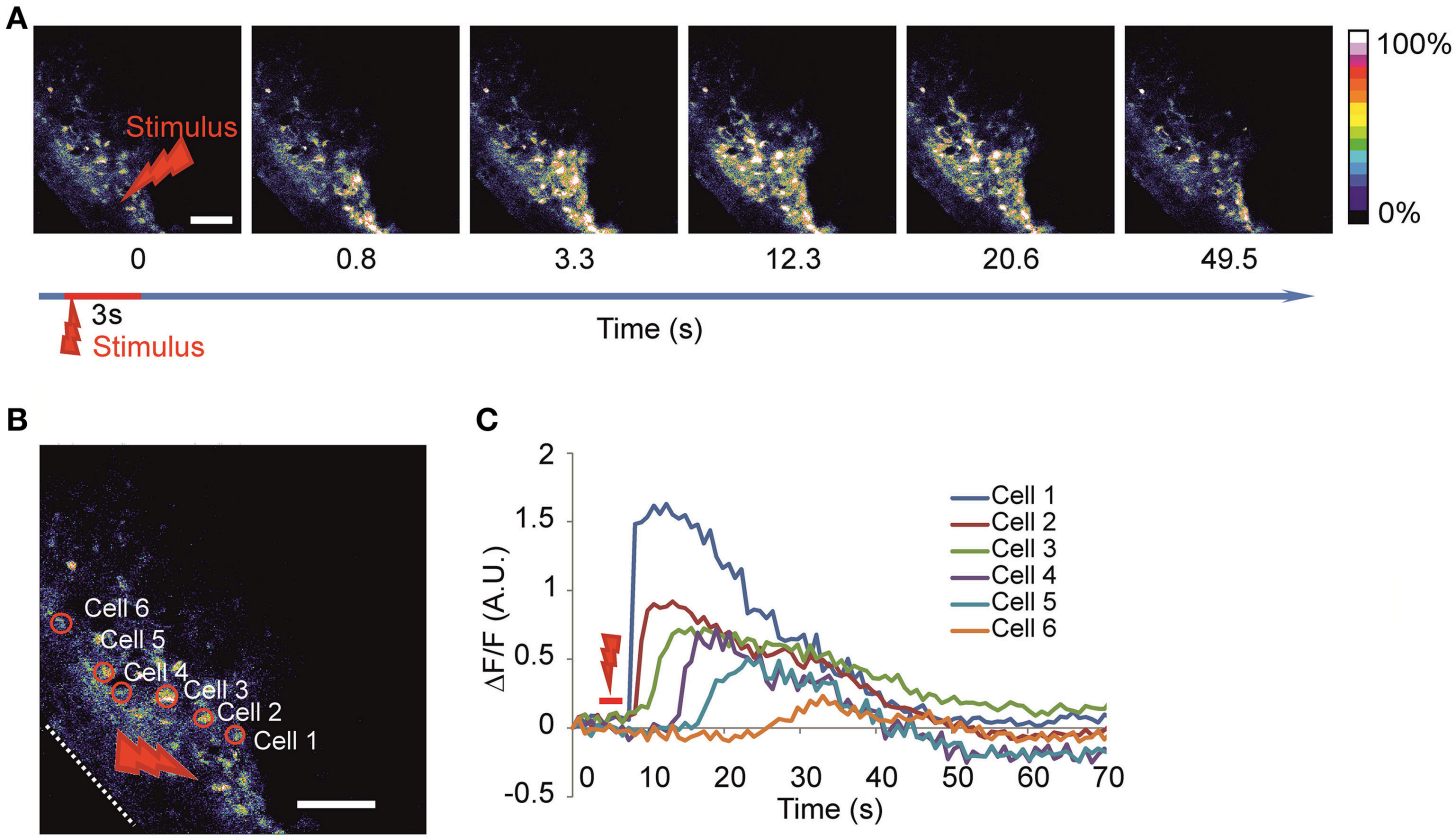
**Imaging of the evoked calcium waves (A) Time-lapse image of calcium wave evoked after laser stimulation of 3 s duration at 100% power (2 W before entering the object) in the round area of 20 μm in diameter. (B)** Enlargement of the same imaged area with examples of identified active cells are delineated on the left panel and the corresponding fluorescence intensity traces **(C)** are shown on the right panel representing the calcium dynamics after stimulation in absolute ΔF/*F*-value. White dotted line delineates the surface of the head. Red arrow indicates stimulation area. Scale bar 100 μm.

We utilized the local high-power laser irradiation paradigm previously used in slices (Liu et al., 2010) and in cultures (Smith et al., 2001) to evoke the calcium waves (Figures 2A–C, Video 1) consistently during the recordings. Using this approach it was possible to evoke calcium waves in response to local laser stimulation. The stability of the specimen was critical for producing localized irradiation. The entire spread of evoked waves could be seen using a 25X objective. To facilitate the comparison of the waves, we normalized the number of active cells to the number and the area of the cells loaded with the calcium dye observed.

### Properties of the calcium waves

**Figure 3:**
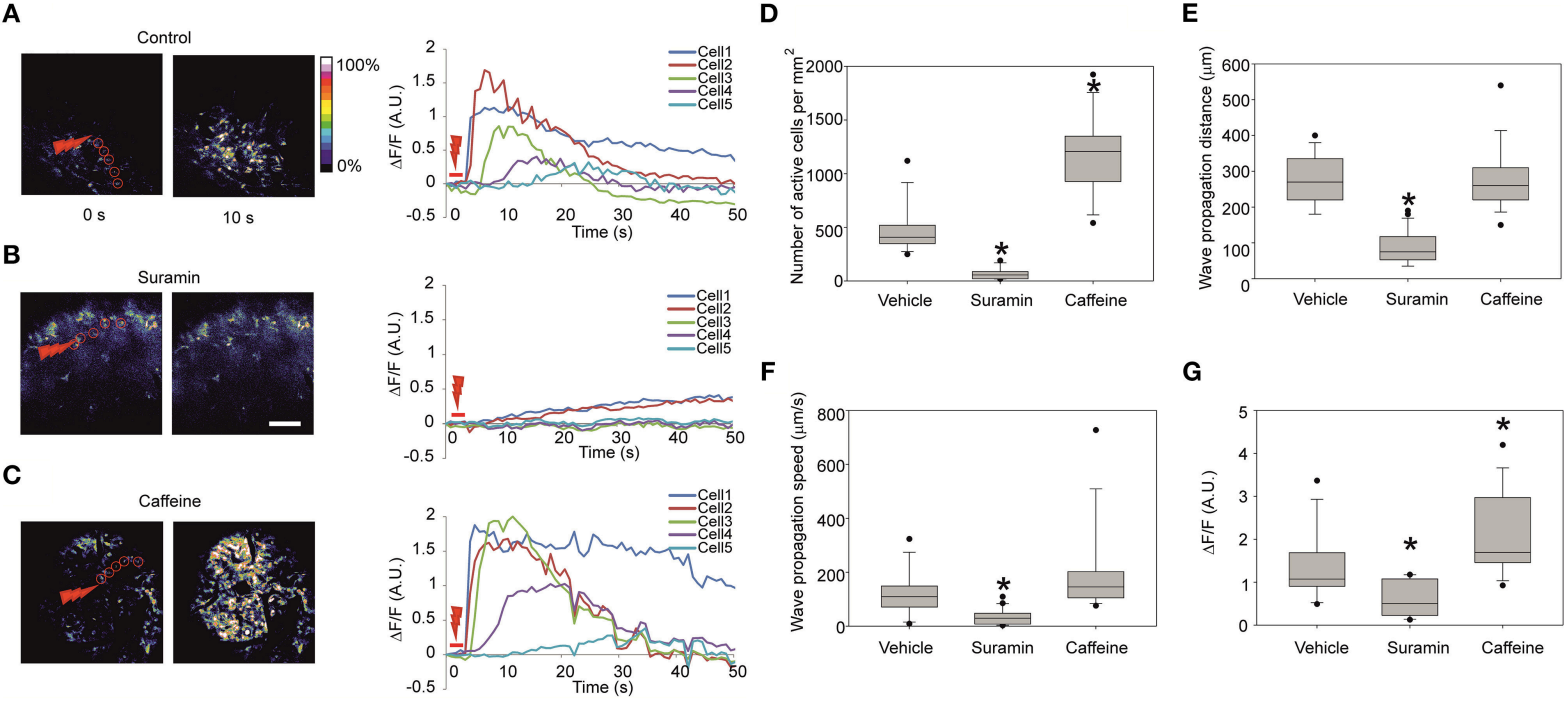
**Representative fluorescence intensity traces under different conditions and quantification of the calcium waves (A) Representative images of the Fluo-4 fluorescence in the cortical area right before stimulation (left) and 10 s after the stimulation (right) and the corresponding fluorescence intensity traces (right panel) in absolute ΔF/***F***-values in control embryos, (B) 60 min after intraventricular injection of the ATP-receptor blocker suramin 3.7 mM**. Note the increase of fluorescence in the cells 1 and 2 indicating successful stimulation. **(C)** Stimulation 20 min after intraventricular injection of caffeine 38.7 mM. Scale bar 100 μm. **(D)** Quantification of the number of active cells involved per mm^2^ area in the visible zone for control vehicle injection (13 recordings in four embryos from two dams), suramin 3.7 mM (16 recordings in three embryos from two dams), and caffeine 38.7 mM (14 recordings in five embryos from three dams) in the stimulated calcium waves. **(E)** Wave propagation distances after injections of suramin and caffeine. **(F)** Wave propagation speeds after injections of suramin and caffeine. **(G)** Fluorescence intensity increase measured at the half-propagation distance after injections of suramin and caffeine in the stimulated calcium waves. Asterisks represent statistically significant difference (*p* < 0.05) in Mann-Whitney *U*-test in comparison to control. Closed circles represent the extreme values of the data sets. Speed is measured as half of the maximum propagation distance divided by the time taken to reach its half-propagation site.

Previously observed calcium waves produced in slices using the laser irradiation were due to a local release of ATP (Liu et al., 2010). To prove the specificity of ATP in producing propagation *in vivo*, we injected 3.7 mM suramin (a non-specific purinergic P2 receptor antagonist) together with the calcium dye at about 1 h prior to laser stimulation to ensure sufficient incubation time. The effect of such treatment resulted in either significantly lower or completely abolished wave propagation as suramin essentially blocked all [Ca^2+^]_i_ responses outside of the stimulus area (Figure 3B; Nunes et al., 2012). The effect is reflected in the decrease of the number of cells recruited in the wave formation of 56 ± 51 cells/mm^2^ (16 recordings in three embryos from two dams) in comparison to 470 ± 220 cells/mm^2^ in control vehicle injection (Figures 3A,D) and in the wave propagation distance of 88 ± 44 μm in comparison to 273 ± 68 μm in control vehicle injection (Figure 3E). The fluorescence increase at the half-propagation distance intensity was reduced by 58 ± 29% (Figure 3G).

To compare wave propagation dynamics under conditions that would facilitate intracellular calcium release from intracellular calcium stores (Friel and Tsien, 1992) we injected 38.7 mM caffeine intra-cerebroventricularly (Figure 3C). 30 min post- caffeine injection the laser-induced calcium-response amplitude of the fluorescence at half-propagation distance increased compared to the control vehicle injection by 55 ± 40% (14 recordings in five embryos from three dams; Figure 3G). Under these conditions additional effects were noted: the total number of cells involved in wave increased to 1190 ± 370 cells/mm^2^ in comparison to 470 ± 220 cells/mm^2^ in control (Figure 3D) and wave propagation speed was increased to 194 ± 163 μm/s in comparison to 119 ± 80 μm/s in control vehicle injection (Figure 3F) though not statistically significant (*p* = 0.093).

**Figure 4:**
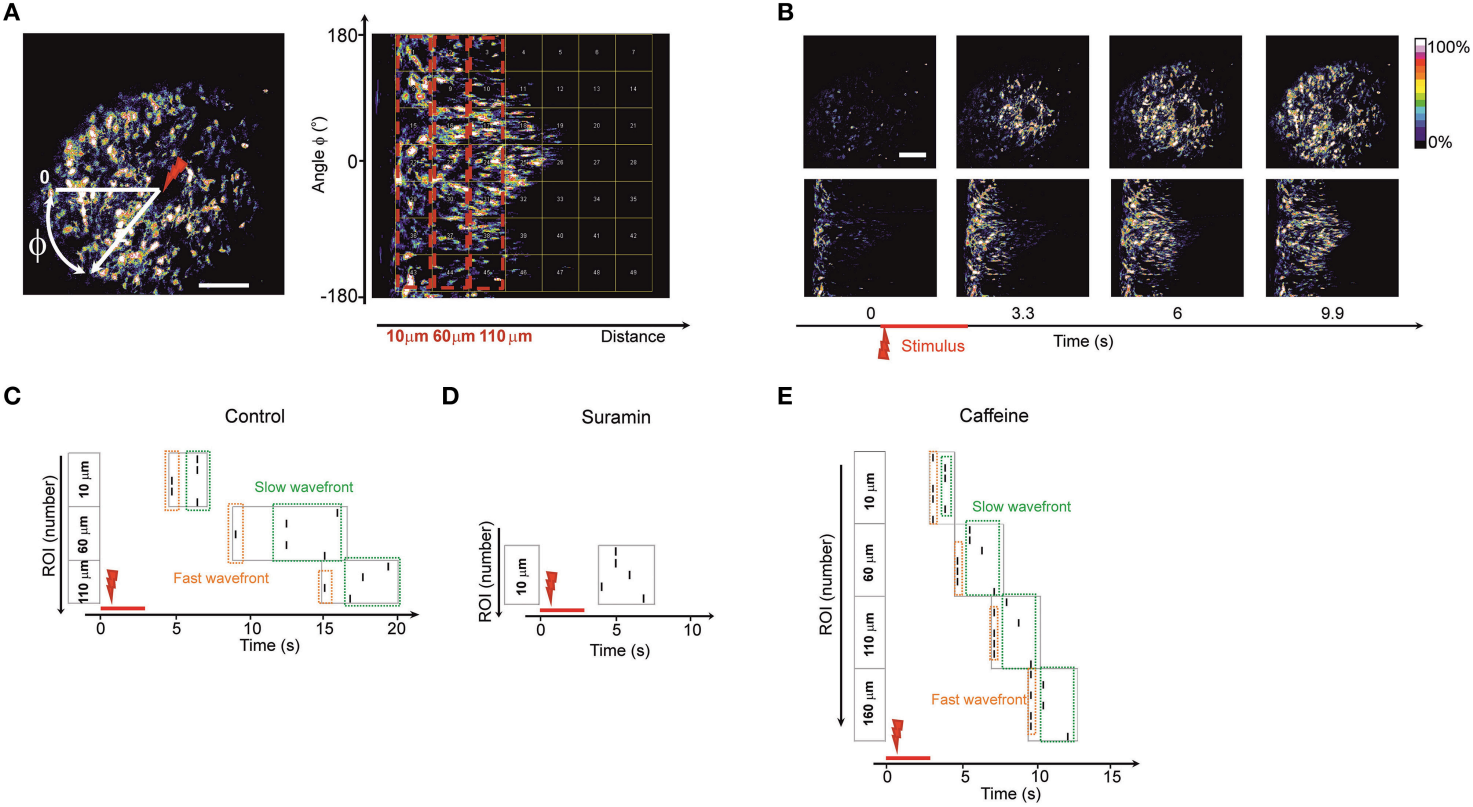
**Wave directionality analysis (A) Polar transformation of the images**. The new matrix (image) is built by transforming the Cartesian coordinates into polar coordinates with a reference point zero (center of transform) as the point of laser stimulus (designated as a red arrow). Therefore, a wave that is propagating in all directions from the stimulation point on the initial image is propagating from left to the right (following the increase of the radial coordinate) on the transformed image. The transformed image is divided into equal-sized regions of interest (square-shaped). Dashed red boxes represent the blocks of regions of interest grouped by their distance from the stimulus point. Regions of interest in the block 10 μm are closest to stimulus center; regions of interest in the block 110 μm are further from the stimulus center. It is possible to analyze the spreading of waves in differing angular directions (vertical coordinate in the transformed image) by following the traces of fluorescence in the polar-transformed images. **(B)** Time-lapse images of the initial image of a wave (upper plane) and time-lapse of the same wave after polar transform (lower plane). **(C–E)** Representative raster plots of events detected in the regions of interest on the polar-transformed images of the waves. The vertical angular coordinate represents the angle of the direction from the observed stimulus point. The events were marked at the time points where the fluorescence intensity exceeded half-maximum of the fluorescent trace in a given region of interest. Dashed boxes represent the events in blocks of regions of interest grouped by their distance from the stimulus point. (Regions of interest in the block 10 μm are closest to stimulus center; regions of interest in the block 110 μm are further from the stimulus center). Dashed green box designates the lagging part of the propagating wave. Dashed orange box designates the leading part of the propagating wave. Similar raster plots were obtained for *N* = 5 of control embryos **(C)**, in embryos 1 h after intraventricular ATP-receptors blocker suramin injection 3.7 mM, *N* = 5 **(D)** and in embryos 20 min after intraventricular caffeine injection 38.7 mM, *N* = 5 **(E)**.

In order to assess whether there are preferences in directionality of the observed waves propagating away from the point of stimulus we decided to transform the image into polar coordinates (Huang et al., 2004) with the stimulus spot as a reference point (Figure 4).

In the polar transformed images waves propagate from left to right along the horizontal axis (Figure 4B). Using this approach it was possible to study the spreading of the waves in all directions and to discriminate if any tracks are faster than others by dividing the transformed image into equal–sized, square-shaped regions of interest (Figure 4A).

In every region of interest we designated an event as being the half-maximum of the fluorescence time trace and built a raster plot (Figures 4C–E). We grouped the regions of interest into blocks of equal distance from the stimulus point. Inside the blocks one could see divergence of events indicating leading and lagging fronts of the calcium wave (i.e., an initial wave, closely followed by a second, more slowly propagating one). In accord with the reduce spread of the wave under suramin the number of blocks observed was smaller than in control condition (Figure 4D). The number of blocks where the wave was detected was typically higher for recordings after caffeine injections since the wave propagation distance was increased (Figure 4E). In every recording situation some tracks were faster than others pointing to an uneven distribution and thus directionality of the wave front.

## Discussion

The results presented here target a dynamic time window in embryonic cortical development (Caviness et al., 1995; Kriegstein and Noctor, 2004). Existing methods for *in vivo* imaging of the embryonic brain have been developed for other species than mammals such as frog embryos (Chang and Spitzer, 2009; Tremblay et al., 2009), zebrafish (Brustein et al., 2003) or chick embryos (McKinney and Kulesa, 2011). Using the isolation of embryos from the dam by a silicone membrane (Pierfelice and Gaiano, 2010) and a fitting heating platform (Caetano et al., 2012) we developed a new approach which fills a critical gap allowing continuous investigation of cortical development *in vivo* under the closest physiological conditions ever achieved so far for intracellular calcium imaging in mammalian embryos. The flexibility of the stimulation procedure allows the assessment of pharmacological intraventricular applications on calcium dynamics. Additionally, these calcium imaging studies can be easily combined with monitoring of cell migration and morphological imaging and eventually *in vivo* electrophysiological recordings.

The soft structure of the embryonic cranium at the early stages of development investigated introduces constraints on the possibilities for tissue stabilization. In some cases it may be challenging to achieve recordings stable enough for cell-by-cell connectivity analysis due to the internal heartbeat of the embryo, especially evident in image disturbances in the deep tissue recordings. However, the approach used for fixation of embryos in the custom-made chamber significantly improved image stability with sufficient quality for performing calcium imaging. The protocol allows quantitative analysis of the calcium signal propagations in different cells populations. It was possible to reliably measure the directionality, speed and intensity of the responses evoked by laser stimulus events.

Using this system it is possible to track the effects of different compounds *in vivo* e.g. acting on the dynamics of intracellular calcium, as shown in the experiments using caffeine. Since calcium is involved in a great variety of intracellular processes during cortex development as well as in adult brains (Ross, 2012), physiological consequences of exposure to different compounds affecting intercellular calcium communications could be evaluated *in situ* and followed by phenotypic analysis at late embryonic stages or postnatal development.

Aside from calcium imaging the monitoring of a great number of other parameters can be envisaged using a similar approach to the one reported. To name just a few: interkinetic nuclear migration, neuronal progenitor migration, and chloride imaging. Thus this method opens a new window of opportunities to investigate the development of the brain under close to physiological conditions.

In the present work we took advantage of the peculiar preference of calcium dye loading to cells in the cortical plate following the intraventricular injection. We identified these cells population to be composed primarily of neurons as imaging experiments on Sox2-GFP mice showed that loaded cells were not positive for Sox2 but were positively labeled by antibodies against neuronal marker Tuj1. Experiments using Sulforhodamine 101 that specifically labels astrocytes (data not shown) confirmed that astrocytes are not present at the imaged developmental time window (Kwan et al., 2012).

To ensure consistent reproduction of the calcium waves and in order to study the properties and underlying mechanism, we used selective stimulations using a two-photon laser irradiation pulse (Liu et al., 2010). Photostimulation has emerged as a useful and versatile approach allowing the induction of localized ATP release, causing consequent calcium wave formation (Smith et al., 2001). For our *in vivo* application it has invaluable advantage over the use of electrodes or mechanical stimulation (Guthrie et al., 1999; Weissman et al., 2004) due to possibility to evoke waves locally with high precision in the deep brain tissues avoiding damages to the brain surface.

Previous *in vitro* data showed that evoked calcium waves in the ventricular zone are sensitive to the activation of ATP-receptors (Weissman et al., 2004). Intraventricular injection of the ATP-receptor blocker suramin strongly suppressed the spreading and amplitude of the waves, further suggesting the involvement of ATP in the propagation of the wave. When calcium release from intracellular calcium stores was facilitated by the injection of caffeine, both the amplitude and number of engaged cells increased significantly.

Analysis revealed a non-uniform spreading of the waves which suggests the presence of different subfamilies of cells with faster and slower propagating signaling, as consistent with the results observed in slices in ventricular zone (Weissman et al., 2004). This may be due to the uneven connectivity through the gap junctions in the immature neuronal ensembles, as previously described in hippocampal slices (Crépel et al., 2007).

## Conclusions

In summary in the present work we have developed a method to monitor intracellular calcium activity in immature neuronal populations in the cortical plate at early embryonic stages *in vivo*. We could evoke the propagating calcium waves in early differentiated neurons using high-power laser irradiation. The evoked waves were suppressed with the blockade of ATP receptors and enhanced with the facilitation of intracellular calcium release. The directionality of the propagating waves could be analyzed differentiating the leading and the lagging wave fronts. The developed method opens the venue of the translational studies in rodent embryos for the disorders connected with network activity malfunctions such as schizophrenia and autism.

## Author contributions

MY, CP, SK, LK, and CR designed the study; MY and VJ developed the imaging system; MY and LA performed the experiments; MY and CR analyzed the data; MY and CR wrote the manuscript.

## Funding

Silgrid Juselius Foundation Helsinki Biomedical Graduate Program.

### Conflict of interest statement

The authors declare that the research was conducted in the absence of any commercial or financial relationships that could be construed as a potential conflict of interest.
